# Efficient and easy‐to‐use capturing three‐dimensional metagenome interactions with GutHi‐C

**DOI:** 10.1002/imt2.227

**Published:** 2024-07-22

**Authors:** Yu‐Xi Lu, Jin‐Bao Yang, Chen‐Ying Li, Yun‐Han Tian, Rong‐Rong Chang, Da‐Shuai Kong, Shu‐Lin Yang, Yan‐Fang Wang, Yu‐Bo Zhang, Xiu‐Sheng Zhu, Wei‐Hua Pan, Si‐Yuan Kong

**Affiliations:** ^1^ Shenzhen Branch, Guangdong Laboratory for Lingnan Modern Agriculture, Key Laboratory of Livestock and Poultry Multi‐Omics of MARA, Genome Analysis Laboratory of the Ministry of Agriculture and Rural Affairs, Agricultural Genomics Institute at Shenzhen Chinese Academy of Agricultural Sciences Shenzhen China; ^2^ School of Life Sciences Henan University Kaifeng China; ^3^ Shenzhen Research Institute of Henan University Shenzhen China; ^4^ College of Informatics Huazhong Agricultural University Wuhan China; ^5^ College of Animal Science and Technology Qingdao Agricultural University Qingdao China; ^6^ State Key Laboratory of Animal Biotech Breeding, Institute of Animal Science Chinese Academy of Agricultural Sciences Beijing China; ^7^ Frederick National Laboratory for Cancer Research Frederick Maryland USA

## Abstract

Hi‐C can obtain three‐dimensional chromatin structure information and is widely used for genome assembly. We constructed the GutHi‐C technology. As shown in the graphical abstract, it is a highly efficient and quick‐to‐operate method and can be widely used for human, livestock, and poultry gut microorganisms. It provides a reference for the Hi‐C methodology of the microbial metagenome. DPBS, Dulbecco's phosphate‐buffered saline; Hi‐C, high‐through chromatin conformation capture; LB, Luria‐Bertani; NGS, next‐generation sequencing; PCR, polymerase chain reaction; QC, quality control.

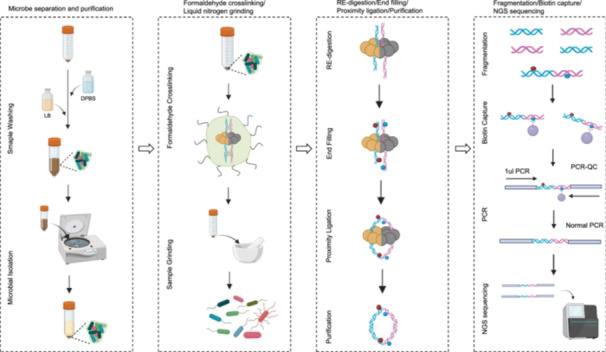

Microorganisms play a key role in ecosystems, and understanding their metagenomic organization is important for understanding the function and interrelationships of microbial populations [1, 2]. Shotgun technology, which is widely used in microbial metagenome research, produces a large number of redundant sequences that can not be classified at the species and strain level [3, 4]. High‐through chromatin conformation capture (Hi‐C) has some obvious advantages in three‐dimensional (3D) organization analysis and genome‐assisted assembly, but it is rarely used in microbes [5, 6, 7]. Therefore, there is an important need for a more efficient and easy‐to‐use Hi‐C technology that can be widely used in human, livestock, or poultry gut microbes. This study introduces a metagenome GutHi‐C technology suitable for microbial populations (Figure 1A). Our method further optimized the experimental conditions, significantly reduced library waste and losses, and conserved experimental reagents (Figure S1). We also created a technical operations video to facilitate the academic exchange of the technology (https://youtu.be/aYEhhRO3eBk). The results show that the quality control parameters of GutHi‐C (such as unique alignment rates, valid data output rates, and valid interaction pair proportion and cis‐interaction ratios) are superior to the data generated by previous methods. GutHi‐C also has a good repeatability (Figure S2). With big data, it exhibited pronounced Hi‐C signal intensity and presented strong chromatin interaction domains, such as chromosomal interaction domains (CIDs) and loop domains. For applications in assembly, assisted by the GutHi‐C, high fidelity (HiFi) platform presents a 38.6% increase in high‐quality metagenomes. Consequently, based on the assessment analysis of GutHi‐C, it would have broad applications in gut microbes of animals, humans, and wide microbial communities, including soil or environmental microorganisms.

**Figure 1:**
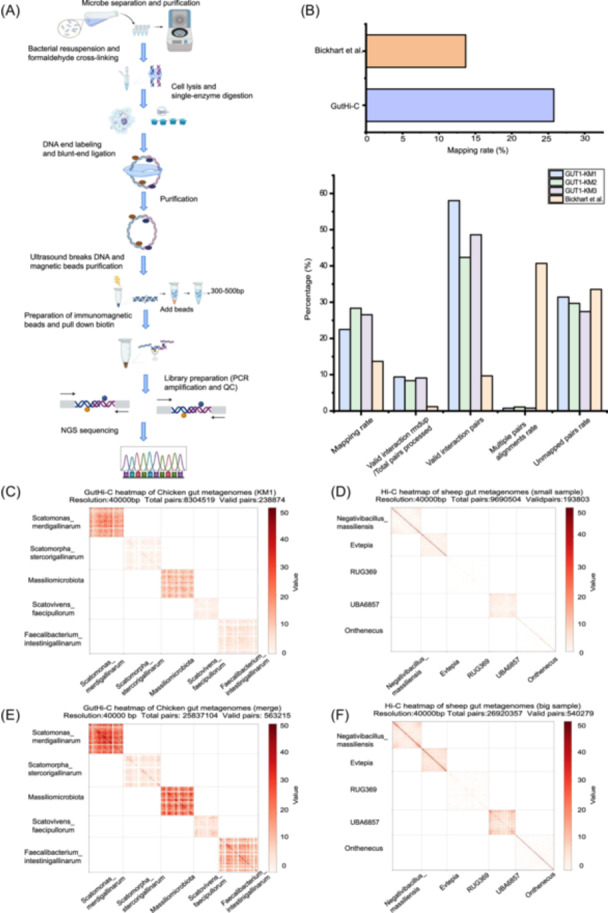
Data evaluation and result analysis for GutHi‐C libraries. (A) GutHi‐C technology roadmap. (B) Evaluation results of two data set comparisons between GutHi‐C and ProxiMeta Hi‐C using HiC‐Pro. (C) Hi‐C heatmap of Chicken gut metagenome (GUT1‐KM1, approximately 8 million total pairs). (D) Hi‐C heatmap of sheep gut metagenome (downloaded, approximately 8 million total pairs). (E) Hi‐C heatmap of Chicken gut metagenome (merge, approximately 25 million total pairs). (F) Hi‐C heatmap of sheep gut metagenome (downloaded, approximately 25 million total pairs). PCR, polymerase chain reaction; QC, quality control; NGS, next generation sequencing; Hi‐C, high‐through chromatin conformation capture.

## DATA EVALUATION OF GUTHI‐C TECHNOLOGY

The library obtained in GutHi‐C was sequenced (approximately 2 gigabases of raw data), and the data were evaluated by HiC‐Pro after processing. The resulting assessments in Figure 1B were compared with the ProxiMeta Hi‐C (also known as Hi‐C Meta) data in the previous work by Bickhart et al. [6]. Compared to ProxiMeta Hi‐C data, GutHi‐C indicates its favorable performance. Its data alignment rate (unique alignment rate), valid pair ratios, and effective data yield rate are comparable to the data generated by the pioneering method [8, 9, 10, 11], which indicates that the GutHi‐C library construction method in this research has more advantages and better quality.

For a more comprehensive comparison, we selected the first five microbial genome interactomes with high assembly quality and alignment enrichment and constructed a Hi‐C heat map for them (Figure 1C,D). We can see that the Hi‐C of metagenomes is mainly concentrated in the interior of genomes, and Hi‐C signals are rarely found outside genomes. We employed interaction frequency heatmaps for comparative analysis of the data set. The test data in this study included GUT1‐KM1, GUT1‐KM2, and GUT1‐KM3. On one hand, compared with GUT1‐KM1 from this study (Figure 1C), the control group was sampled to approximately 8 million total pairs (Figure 1D). On the other hand, compared with the combined data of GUT1‐KM1, GUT1‐KM2, and GUT1‐KM3 from this study (Figure 1E), the control group was sampled to approximately 25 million total pairs (Figure 1F). Comparative analysis reveals that, under equal data volume conditions, the methods used in GUT1‐KM1 (Figure 1C) significantly outperformed the control group (Figure 1D). Furthermore, the interaction frequencies of the combined data of GUT1‐KM1, GUT1‐KM2, and GUT1‐KM3 (Figure 1E) also surpassed those of the control group (Figure 1F). Even if the initial assembly quality of the reference genome is not good, more interactions are produced. The GutHi‐C technology can still produce higher *cis*‐interaction (intra‐microorganism) ratios. It would have great application potential in the complete metagenome‐assisted assembly in the future.

## APPLICATION OF GUTHI‐C TO REVEAL THE 3D CONFORMATION OF MICROBIAL METAGENOMES

**Figure 2:**
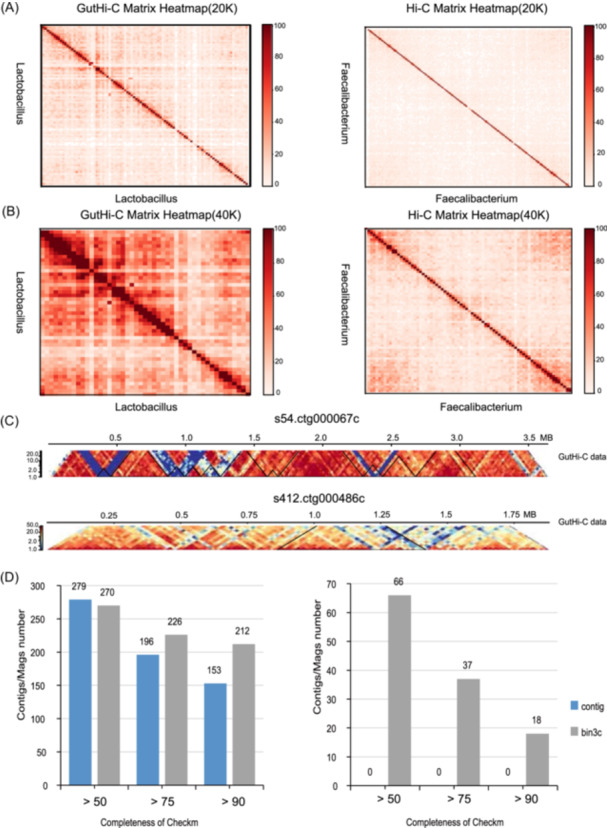
Assessment analysis and preliminary application to reveal the 3D conformation of microbial metagenomes and assist metagenome assembly. (A) High‐through chromatin conformation capture (Hi‐C) matrix heatmap of the gut microbiota by GutHi‐C at a resolution of 20 kb bin (left). The Hi‐C matrix heatmap of the gut microbiota by ProxiMeta Hi‐C at a resolution of 20 kb bin (right). (B) Hi‐C matrix heatmap of the gut microbiota by GutHi‐C at a resolution of 40 kb bin (left). The Hi‐C matrix heatmap of the gut microbiota by ProxiMeta Hi‐C at a resolution of 40 kb bin (right). (C) Chromosomal interaction domain (CID) and local interaction of the GutHi‐C map (20 kb bin resolution) in some bacterial strains in the chicken gut. (D) Evaluation of the high fidelity (HiFi) assembly from Zhang et al.'s chicken gut data set, assisted by GutHi‐C based on hifiasm‐meta (left). Evaluation of the assembly in the illuminal data set, assisted by GutHi‐C based on spades. The blue bar and gray bar represent the draft assembled contigs and the metagenome‐assembled genomes (MAGs) assisted by GutHi‐C using bin3c, respectively. The results generated by CheckM2 and all contamination of contigs/MAGs were less than 10% (right).

We have recollected the gut microbiota of the experimental chickens and reconstructed the GutHi‐C libraries, which were submitted to large‐scale sequencing (approximately 100–150 gigabases of raw data). We selected the top 10 single bacteria from both our method and the control group ProxiMeta Hi‐C for heatmap comparison. This comparison revealed that our method exhibited a more pronounced signal intensity, as illustrated in Figure S3A–3B. Subsequently, we zoomed in on the bacteria with the strongest signal and showed higher‐resolution plotting of the assembled and aligned genomes. It can be observed from both 20 kb bin and 40 kb bin resolutions that our method exhibits significantly stronger signal intensity (local interactions, also known as loops) (Figure 2A,B). Additionally, there are interactions in specific local regions within a single bacterial strain. As illustrated in Figure 2C, the region highlighted by the solid black triangle in the diagram represents an area of topologically associated domain (TAD)‐like strong interact frequency region, called CIDs. Our results indicate that there are regions within individual bacteria in the GutHi‐C that represent strong interactive patterns. In contrast, the Hi‐C heatmap of ProxiMeta Hi‐C shows no apparent presence of interaction regions under a similarly large number of data conditions.

## APPLICATION OF GUTHI‐C TO ASSIST METAGENOME ASSEMBLY COMBINED WITH HIFI THREE‐GENERATION SEQUENCING

Hi‐C for gut microbiota has not yet been widely applied to assist microbial completed (ring‐forming) metagenome assembly. Most applications have been limited to assisting in eukaryotic genome assembly or 3D conformation analysis [12, 13, 14, 15]. We conducted extensive sequencing on samples from the cecum of the chicken gut microbiome in our GutHi‐C library construction method. We used samples (such as Gut1) with higher alignment rates to assist assembly and compare with the previous assembly results of the chicken gut metagenome in Zhang et al. (Zhang et al. previously obtained a third‐generation sequencing‐assembled chicken gut metagenome as a reference genome) [16]. In our research, the assembly results of chicken's third‐generation HiFi sequencing were assisted by GutHi‐C and binned using bin3C [17]. Simultaneously, we also binned the homologous metagenome samples of Illumina sequencing data. The results, as shown in Figure 2D, demonstrate that owing to the good quality of GutHi‐C data combined with the high accuracy of HiFi, we obtained 212 high‐quality metagenome‐assembled genomes (MAGs) (Completeness > 90 and Contamination < 10) and 226 MAGs of medium to high quality (Completeness > 75 and Contamination < 10).

This represents a 38.6% increase in high‐quality genomes compared to the previous contig‐level assemblies. However, the number of medium‐quality MAGs (Completeness > 50 and Contamination < 10) has decreased. This suggests that using our Hi‐C data allows for the classification of low‐quality or medium‐quality contigs, thereby enhancing the assembly quality of high‐quality HiFi metagenomes. In Figure 2D, for enhancing the assembly quality of next‐generation sequencing (NGS) shotgun sequencing metagenomes by GutHi‐C, the difference is more apparent. In the initial assembly of contigs using Illumina TruSeq Shotgun sequencing, none achieved MAGs of medium quality (0 MAGs), medium to high quality (0 MAGs), and high quality (0 MAGs). However, with the corresponding GutHi‐C data set, we obtained 66 medium‐quality MAGs (Completeness > 50 and Contamination < 10), 37 medium to high‐quality (Completeness > 75 and Contamination < 10), and 18 high‐quality MAGs (Completeness > 75 and Contamination < 10).

## DISCUSSION

The technical advantages of the GutHi‐C will be discussed in terms of the following, in the order of technical steps. Microbial lysis is carried out by liquid nitrogen grinding and lysozyme treatment. As a result, the microbial cell wall could be permeabilized to the maximum extent, and the DNA material could be fully accessible. Thus, subsequent restriction endonucleases can thoroughly fragment the microorganism's genome, and ligases can effectively access and produce good ligation within the nucleoid region, thereby ensuring higher efficiency throughout the subsequent library construction steps. It achieved data with a higher valid interaction pair proportion and valid data output rate. Besides, microbial lysis using only lysozyme treatment reduces library loss and significantly increases processed DNA concentration, making it suitable for small microbial populations. Liquid nitrogen grinding may lose a small number of microbial samples. Hence, when microorganism quantity is minimal, lysozyme lysis alone could be performed to obtain high DNA concentrations as needed. During the library construction process of GutHi‐C, the introduction of the “in situ Hi‐C framework” could retain the original microenvironment of the nuclear region, make the proximity‐ligation be carried out in the nuclear region to the greatest extent, improve the ligation efficiency, and lower background noise compared with traditional Hi‐C [18]. Additionally, the proximity‐ligation reaction solution used in this study contains recombinant albumin with easier access, which can replace BSA in existing technologies and serves the same function. In this study, biotin is served as a blunt end marker, but with the dosage being only half of that required for a conventional in situ Hi‐C system, maintaining a good outcome. In other words, this not only maintains or enhances the original effect but also reduces the cost by halving the usage of the most expensive biotinylated reagent in the technical steps. GutHi‐C uses T1 immunomagnetic beads for library capture. The quantity of T1 beads has been reduced threefold, from 150 μL [13] to 50 μL, while still maintaining library construction efficiency (Figure S1). This contributes to a continued reduction in experimental costs. In addition, the method put the chimeric interaction DNA enrichment step before the NGS A‐tailing and adapter addition library construction, so that the reagent consumption is greatly reduced. Moreover, the polymerase chain reaction quality control (PCR‐QC) test is carried out before DNA formal amplification. It can obtain the optimal amplification conditions, improve the preparation ratio of the GutHi‐C library, and avoid reagent waste. Importantly, it could significantly reduce library loss. Concretely, Micro‐library is just introduced for preamplification. These advantages would be the reasons that make GutHi‐C's results superior to current technologies. In addition, by setting up different experimental variables for comparison, the results demonstrate that GutHi‐C has good repeatability (Figure S2).

There are currently few literature reports on the metagenome Hi‐C data of chicken intestinal microbes. On the one hand, we could not download the corresponding control group data; on the other hand, there are few well‐established and available microbial metagenome Hi‐C technologies or kits that can be used. For instance, detailed methods or kits for microbial Hi‐C currently available in the industry are not accessible in domestic regions. We cannot get it through formal means. Meanwhile, we learned that the kits are very expensive, about $1800–2500 (e.g., ProxiMeta Hi‐C [Phase Genomics] kit); however, the cost of GutHi‐C is only $400–600. The restricted sales and high cost of the ProxiMeta Hi‐C are also significant factors for motivating us to develop the efficient and easy‐to‐use GutHi‐C technology. Therefore, we can only download the reported ProxiMeta Hi‐C data as control groups, such as the intestinal microbiome Hi‐C data of sheep and cows prepared by ProxiMeta Hi‐C technology. Moreover, GutHi‐C exhibited stronger Hi‐C signal intensity and presented strong chromatin interaction domains compared to the existing Hi‐C (Figure 2A‐C and Figure S3). Meanwhile, in the supplemental material, we supplemented and comprehensively evaluated the results and superiority of GutHi‐C technology in detail. We also compared our experimental procedure with the manual from the ProxiMeta kit that was downloaded from the Phase Genomics website (Table S1).

An important application direction for GutHi‐C is metagenomic‐assisted assembly. ProxiMeta Hi‐C was assisted in assembling metagenomes of the rumen microorganisms of cow, which reduces the amount of sequencing and sampling required. In these two research [4, 9, 10], researchers used assembly strategies for Hi‐C coupled with second‐generation Illumina TruSeq Shotgun sequencing [9, 10] and Hi‐C coupled with third‐generation PacBio RS SMRT sequencing [10], respectively. As known, the read length of Illumina Shotgun is always as short as 150–250 bp and the error rate of PacBio SMRT is very high (~15%) [19]. As a consequence, these may lead to folded repetitive sequences and the loss of regions that do not assemble well at all due to their complexity. This study has another important significance. We also present two good applications in the metagenome assembly for GutHi‐C. On the one hand, it was proved that Hi‐C combined with next‐generation Illumina sequencing significantly increased the number of ring‐forming microbial genomes. On the other hand, we confirmed the feasibility of Hi‐C‐assisted assembly of high‐quality HiFi reads to obtain more ring‐formed intact metagenomes with high fidelity.

Therefore, we suspect that GutHi‐C technology can have an impact and application range comparable to that of other current technologies.

## AUTHOR CONTRIBUTIONS

**Si‐Yuan Kong**: Conceptualization; funding acquisition; investigation; methodology; project administration; supervision; validation; visualization; writing—original draft; writing—review & editing. **Wei‐Hua Pan**: Conceptualization; resources; software; supervision. **Yu‐Xi Lu**: Formal analysis; investigation; validation; visualization; writing—original draft. **Jin‐Bao Yang**: Data curation; formal analysis; methodology; software; visualization. **Chen‐Ying Li**: Investigation; validation. **Yun‐Han Tian**: Investigation; visualization. **Xiu‐Sheng Zhu**: Resources; funding acquisition; writing—review & editing. **Rong‐Rong Chang**: Validation; writing—review & editing. **Da‐Shuai Kong**: Project administration; writing—review & editing. **Yu‐Bo Zhang**: Supervision. **Shu‐Lin Yang**: Funding acquisition; supervision. **Yan‐Fang Wang**: Supervision.

## CONFLICT OF INTEREST STATEMENT

The authors declare no conflict of interest.

## ETHICS STATEMENT

The ethics application (No. AGIS‐ER‐2024‐018) was approved by the Life Science Ethics Committee of the Agricultural Genomics Institute at Shenzhen, Chinese Academy of Agricultural Sciences, and adhered to China's microbial control, animal ethics, and animal welfare requirements.

## Supporting information


**Figure S1.** Gel plots for the quality control during library construction.
**Figure S2.** Correlation of GutHi‐C matrices under different experimental conditions.
**Figure S3.** Different resolution hierarchical structures of the single‐bacterial metagenome revealed by GutHi‐C, compared to ProxiMeta Hi‐C.

**Table S1.** Comparison of the GutHi‐C experimental procedure with the manual from this kit that was downloaded from the Phase Genomics website.

## Data Availability

These sequence data have been submitted to the CNCB (https://ngdc.cncb.ac.cn/bioproject/) databases under accession number PRJCA026342 including three submissions with the accession number CRA016650 (Gut5 Hi‐C), CRA016599 (Gut1 Hi‐C), and CRA016732 (Gut1 WGS) in GSA (https://ngdc.cncb.ac.cn/gsa/). The data of assembly can be accessed at http://ftp.agis.org.cn:8888/~panweihua/chicken/. The data and scripts used are saved in GitHub (https://github.com/ruoyu1123/Gut-HiC). Supplementary materials (introduction, methods, results, discussion, figures, tables, scripts, graphical abstract, slides, videos, Chinese translated version, and updated materials) may be found in the online DOI or iMeta Science http://www.imeta.science/.
