# Supplementary material for: Efficient and easy‐to‐use capturing three‐dimensional metagenome interactions with GutHi‐C
Source: Imeta. 2024 Jul 22;3(5):e227. doi: 10.1002/imt2.227 (PMC11487548; doi:10.1002/imt2.227)
Supplement: Supplementary file 1 — Figure S1. Gel plots for the quality control during library construction. Figure S2. Correlation of GutHi‐C matrices under different experimental conditions. Figure S3. Different resolution hierarchical structures of the single‐bacterial metagenome revealed by GutHi‐C, compared to ProxiMeta Hi‐C. [file IMT2-3-e227-s001.docx]

# Supporting information to

# Efficient and easy-to-use capturing 3D metagenome interactions with GutHi-C

**Running title:** Capturing 3D metagenome interactions with GutHi-C

Yu-Xi Lu^1,2,3#^, Jin-Bao Yang^1,4#^, Chen-Ying Li^1,5#^, Yun-Han Tian^1,5^, Rong-Rong Chang^1,2,3^, Da-Shuai Kong^1,2,3^, Shu-Lin Yang^6^, Yan-Fang Wang^6^, Yu-Bo Zhang^7^, Xiu-Sheng Zhu^1^*, Wei-Hua Pan^1^*, Si-Yuan Kong^1^*

^1^Shenzhen Branch, Guangdong Laboratory for Lingnan Modern Agriculture, Key Laboratory of Livestock and Poultry Multi-omics of MARA, Genome Analysis Laboratory of the Ministry of Agriculture and Rural Affairs, Agricultural Genomics Institute at Shenzhen, Chinese Academy of Agricultural Sciences, Shenzhen 518120, China.

^2^School of Life Sciences, Henan University, Kaifeng 475004, China.

^3^Shenzhen Research Institute of Henan University, Shenzhen 518000, China.

^4^College of Informatics, Huazhong Agricultural University, Wuhan 430070, China.

^5^College of Animal Science and Technology, Qingdao Agricultural University, Qingdao 266109, China.

^6^State Key Laboratory of Animal Biotech Breeding, Institute of Animal Science, Chinese Academy of Agricultural Sciences, Beijing 100193, China.

^7^Frederick National Laboratory for Cancer Research, 8560 Progress Drive, Frederick, MD 21701, USA.

^#^These authors contributed equally: Yu-Xi Lu, Jin-Bao Yang, Chen-Ying Li

*Correspondence: kongsiyuan@caas.cn (Si-Yuan Kong), panweihua@caas.cn (Wei-Hua Pan), zhuxiusheng@caas.cn (Xiu-Sheng Zhu).

**Supplemental Material Contents**

**Supplemental** **Introduction**

**Experimental Design**

Overview of the procedure

**Protocol of the GutHi-C**

Isolation of gut microbiota

The microorganism was cross-linked with formaldehyde

Efficient cell lysis, permeabilization and fragmentation digestion

Biotin-labeled end-filling and blunt-end circularization ligation

Reversal cross-linking to obtain purified DNA

Circular coprecipitated DNA was introduced and the joint DNA was disrupted by sonication

Magnetic beads sorting purification

The target interaction fragments were captured by immunomagnetic beads

End repair and A-tailing reactions was performed and the library was amplified and sequenced

**Supplemental Result**

Data evaluation and result analysis for GutHi-C libraries, which were sequenced in small size data

Big data size sample assessment analysis and preliminary application to reveal the 3D conformation of microbial metagenomes

**Supplemental Discussion**

**Data Available**

**References**

**Supplemental Figure**

Figure S1

Figure S2

Figure S3

**Supplemental Introduction**

The high-throughput chromosome conformation capture (Hi-C) approach was developed in 2009 [1] and has greatly expanded the understanding of 3D genomics [2−4]. The Hi-C technology is based on the ''Proximity Ligation'' core step of 3C, which combines with high-throughput sequencing and can capture genome-wide specific and non-specific chromatin interactions. Through bioinformatics analysis, one-dimensional interaction linkage maps, two-dimensional interaction heat maps, regulatory networks, and three-dimensional structural information can be studied [1,5]. Genome assisted genome assembly [6,7] can also be performed with Hi-C. Hi-C has many derivatives, including *in situ* Hi-C [8], Single-cell Hi-C [9], Capture-Hi-C [10], DNase Hi-C [11], DNase capture-Hi-C [11], and scNanoHi-C [12].

To date, Hi-C technology has been applied to a wide range of species. In a chicken erythroblast cell line HD3, Hi-C allowed assessment of spatial genomic organization and chromosomal aberrations in an avian karyotype with a large number of microchromosomes [13]. By integrating GWAS (Genome-Wide Association Studies) and epigenomics data, including ChIP-seq (Chromatin Immunoprecipitation Sequencing), associating with porcine LMD (medial ligament muscle depth), a QTL (quantitative trait loci) and its candidate genes within this region were identified [14]. And an *in situ* Hi-C protocol suitable for compatibility with the BGISEQ-500 high-throughput sequencing platform was developed in peacock fish tapeworm [15]. In addition to this, Hi-C technology also can be used to study the network of chromatin interactions in plants [16]. In *Arabidopsis thaliana*, chromatin remodeling complexes were found to regulate 3D chromatin structure and gene expression [17] by *in situ* Hi-C. Other study has shown that Hi-C can reveal extensive hierarchical chromatin interactions in rice [18]. Moreover, the Hi-C technology has evolved over time and the upgraded Hi-C technology has been tested in other plant samples, such as leaves of soybeans, where the upgraded Hi-C technology is a more efficient option compared to the conventional Hi-C [19]. Despite the widespread application of Hi-C technology in mammals, as well as in plants, there has been some limitations in the microorganism [20].

Gut microbiota is a complex microbial community associated with human health and disease [21,22]. Due to the importance of the intestinal microbiota, the composition and role of microorganisms have been intensively studied in recent years. Currently, there are two main methods for the identification of human and animal gut microbes, including 16S rRNA sequencing and Shotgun sequencing. 16S rRNA sequencing can be used to analyze the intestinal flora of human and animal at a low cost. 16S rRNA sequencing does not retrieve genomic information, whereas Shotgun sequencing can. For example, 460 novel microbial strains were collected from the chicken cecum microbiota. Through genome sequencing, assembly and sorting, these data would form the basis for future studies on the structure and function of the chicken intestinal microbiota [23]. Besides, metagenomic sequencing of collected fecal samples from Tibetan pigs to investigate unknown microbial species in the gut and possible genomic biomarkers in the microbiome [24]. However, the Shotgun techniques not only produce a large number of short redundant sequences that cannot be categorized at the species and strain level [25], but also result in wasted sequencing data resources and loss of genomic information for many strains. Over time, three generation sequencing (TGS) technology facilitate the study of microbial communities. Studies show that the prokaryotic genome was preliminarily obtained and researched by using the HiFi sequencing [26].

If Hi-C technology is further applied in metagenomic assisted assembly, similar to the assembly method of eukaryotic genomes, better results would be achieved. For instance, a combination of hybridization sequencing (Illumina Shotgun and ONT) and Hi-C technologies characterized 109 gut metagenome and identified 70 previously uncharacterized gut microbial species in the human gut of Southeast Asian populations [27]. For the rumen metagenome research of 43 Scottish cattle, with Hi-C and HiFi assembly, it was demonstrated that the identified microbes play an important role in animal physiology and disease [28]. But current published Hi-C technologies used in these studies involving animals and plants cannot be directly applied to microorganisms. Currently published microbial Hi-C data quality is suboptimal, and the relevant literature has not disclosed specific operational details and compositional information regarding microbial Hi-C [25,27,29−31]. And microbial Hi-C studies often yield small datasets, resulting in less homogeneous and reproducible data. Additionally, the strong cell walls of microorganisms make them challenging to lyse, rendering Hi-C techniques ineffective. Therefore, there is a need for a more efficient and easy-to-use method, specifically designed for microorganisms. Thus we constructed the GutHi-C technology that improves the quality of library construction and sequencing data while reducing experimental costs for a wider range of applications.

##

## Experimental Design

### Overview of the procedure

This protocol aims to provide a metagenomic GutHi-C library-building method and application for microbial populations. which including the following steps: 1) microbial separation and purification: the microbial sample is washed, the culture medium is added and naturally settled, and the microbe is separated by centrifugal force; 2) crosslinking, permeabilization and dissociation: microbial cells were cross-linked with 37% formaldehyde, then cell walls were disrupted by grinding with liquid nitrogen and incubating by lysozyme; 3) efficient digestion, end-filling and proximity ligation: the product is digested by RE-enzyme (restriction endonuclease), and then the adhesive end is supplemented by a biotin label, and the interactive DNA is adjacent ligated; 4) Chaperone DNA addition and sonication: the product is purified, and the circular coprecipitated DNA is added, and the interactive DNA is ultrasonic fragmented; 5) Biotin enrichment and adapter addition: basing on the biotin enrichment of adjacent interaction fragments, the end is repaired and are connected with the linker on the immunomagnetic beads; 6) PCR and NGS-sequencing: the quality control test of library pre-amplification is carried out, and the optimal amplification conditions selected by the test are officially amplified to obtain the GutHi-C library. Finally, the GutHi-C library is submitted to BGI or Illumina sequencing. The data analysis softwares of GutHi-C are simillar to the standard Hi-C processing pipelines and Hi-C-assisted genome assembly softwares, such as Cutadapt [32], HiC-Pro [33], HiCExplorer [34], bin3C [35], CheckM2 [36] and so on [37,38].

## Protocol of the GutHi-C

The technical route of microbial metagenome GutHi-C technology is shown in Figure 1A. We also have created instructional videos illustrating the experimental procedures to provide a more intuitive understanding for readers to use the GutHi-C technology.

### Isolation of gut microbiota

This protocol takes the gut microbes of Chinese Lingnan yellow chickens as the research object, to perform metagenomic GutHi-C protocol, the following steps were performed. The initial step of this experimental protocol involves the microbial washout process, aimed at extracting microbial communities as thoroughly as possible from the intestinal contents. After sample washing, 12000 × *g* centrifugation was performed.

### The microorganism was cross-linked with formaldehyde

The treated samples were washed and precipitated with DPBS, followed by adding 37% formaldehyde for cross-linking, 2.0 M glycine for de-cross-linking, centrifuging at room temperature for 5 min at 2000 × *g*. The samples was then followed by the addition of 3 mL of precooled 1 × DPBS resuspended cells, with each 1 mL sample packed in a 1.5 mL centrifuge tube (EP tube) (actual 0.166 g), centrifugation at 4°C 2000 × *g* for 5 min and aspiration of supernatant. Then the samples were put in -80° C refrigerator or carried on the next step.

###

### Efficient cell lysis, permeabilization and fragmentation digestion

Parallel lysis of microbial cell walls by liquid nitrogen grinding and lysozyme was operated. Each sample was divided into 3 groups (sample nomenclature: Take GUT1-KM1 as an example, GUT represents intestinal tract, 1 represents test batch, K represents test personnel, M represents microorganism of the cecal segment, 1 represents sample number under all batches), group 1 is named GUT1-KM1, and the treatment is lysing microbial cell wall by liquid nitrogen grinding and lysozyme chemical incubation in continuous operation. They should be rapidly ground to a powder state. Group 2, named GUT1-KM2, is treated only by grinding with liquid nitrogen. The third group, named GUT1-KM3, is only treated with lysozyme.

Then it was centrifugated at 25°C 2000 × *g* for 5 min. The supernatant is aspirated, and the sample was resuspend by adding 500 μL of 1× NEBuffer™ DpnII (NEB R0543S). Each tube was added restriction enzyme with 5~20 μL (DpnII, Neb R0543s, 10,000 units mL) at 37°C for overnight.

### Biotin-labeled end-filling and blunt-end circularization ligation

The samples treated in step 1.3 were incubated at 62°C for 20 min, and centrifuged at 2000 × *g* room temperature (RT) for 5 min. The supernatant was discarded. 265 μL of 1× Cutsmart buffer (NEB B6004) was added and mixed well. Then, 25 μL of fill-in master mix and 25 μL of 1× Cutsmart buffer were added and mixed well. In dilution Hi-C, *in situ* Hi-C and other conventional Hi-C systems, 50 μL of fill-in master mix is required. But in this protocol, only 25 μL of fill-in master mix is needed. As the price of Cutsmart is cheaper than biotin, it saves the cost of building a library. The fill-in master mix should be prepared according to the standard procedure in the total volume of 50 μL, the components are as follows: 1.5 μL 10 mM dATP (Thermo Fisher), 1.5 μL 10 mM dGTP (Thermo Fisher), 1.5 μL 10 mM dTTP (Thermo Fisher), 37.5 μL 0.4 mM biotin-14-dATP (Thermo Fisher), and 8 μL 5 U/μL Klenow (NEB). The sample was placed at water bath for shaking incubation in 37° C for 1.5 h. Then, 900 μL of ligation master mix (120 μL 10 × T4DNA ligase buffer (NEB), 100 μL 10% Triton X-100, 6 μL 20 mg/mL Recombinant Albumin (NEB), 5 μL 400 U/μL T4 DNA Ligase (NEB), and 669 uL ultrapure water) was added. We mixed them by inversion and rotation at RT for 4 h.

### Reversal cross-linking to obtain purified DNA

50 μL of Proteinase K (20 mg/mL) and 120 μL of 10% SDS were added and incubated at 55°C for 30 min. 130 μL of 5 M sodium chloride was added and incubated at 68°C overnight, then the test tubes were placed at RT, and the samples were split into 750 μL and moved into new centrifuge tubes. Respectively, 1.2 mL of absolute ethanol and 75 μL of 3 M sodium acetate (pH 5.5) were added, mixed upside down, and incubated at -80°C for 15 min. Then they were centrifugated at 2°C 21000 ×*g* for 15 min after incubation. After centrifugation, they were immediately put on ice and their supernatant was discarded. The same sample was washed by 400 μL of 80% ethanol twice. Following by combining 800 μL ethanol with sample, it was transferred into a new centrifuge tube and centrifuged at 4°C 21000 ×*g* for 10 min. The supernatant was then dried in a 4°C refrigerator for 1~3 h. After resuspending in 131 μL of 1 × Tris buffer (10 mm Tris-HCl, pH 8) and incubating at 37°C for 20 min, it was centrifuged at 3000 g for 1.5 min at RT, and transferred into a new centrifuge tube, DNA concentration was measured using Qubit. For example, the concentration of GUT1-KM1 was 55.6 ng/μL, the concentration of GUT1-KM2 was 82.2 ng/μL, and the concentration of GUT1-KM3 was 90 ng/μL. As GUT1-KM3 were treated with lysozyme only, the highest concentration of purified DNA was obtained, without trituration.

### Circular coprecipitated DNA was introduced and the joint DNA was disrupted by sonication

1.95 μL of circular coprecipitated DNA (~200 ng to observe the interruption effect as a control) was add to the reversal cross-linking library DNA of the formal part. 1 × Tris buffer (pH 8.0) should be added for filling the solution to 130 μL. The solution was mixed well and transferred to a microTUBE tube (Covaris 220). The total circularized DNA was interrupted according to the program (Fill Level 10, Duty Cycle 15, PIP 500, Cycles per Burst 200, Time 58 s). All samples from the microTUBE were transferred to new centrifuge tubes. Ultra-pure water was needed to further wash the microTUBE tube, and transferred them to the library, the volume of the library solution should be aligned to 200 μL. 2 μL of DNA solution was run on 2% E-gel, and the results are shown in Figure S1A. The results show that the effect of somnication interruption is ideal. 2 μL of ultra-pure water was added up to 200 μL, which is the starting volume of the library for magnetic beads sorting.

### Magnetic beads sorting purification

Magnetic bead sorting (300~500 bp) was performed after ultrasound interruption. Firstly, Ampure XP bead solution was put at RT before use. Secondly, 110 μL of Ampure XP beads were added to each sample (0.55% of the starting volume 200 μL of the library). It was pipetted, mixed and incubated for 5 min at RT. The samples were put on a magnetic rack for 5 min. Then, the supernatant was transferred to a new centrifuge tube, meanwhile the operator should avoid pipetting magnetic beads. 30 μL of new Ampure XP beads were added (0.15% of the starting volume of the library). It was pipetted, mixed, and incubated for 5 min at RT. Then it was placed on the magnetic rack for 5 min, After the supernatant was removed, the magnetic beads with DNA were retained and washed with 700 μL of 80% alcohol twice. Then they were placed back on magnetic rack for 5 min for evaporating the alcohol. For elution of DNA, 300 μL 1 × Tris Buffer was added. The solution was gently pipetted, mixed and incubated for 5 min at RT. The tubes were placed on the magnetic rack for 5 min, and the supernatant was transferred to a new 1.5 mL centrifuge tube. The concentration was determinated by using Qubit kit. For example, the concentration of GUT1-KM1 was 1.3 ng/μL. The concentration of GUT1-KM2 was 2.72 ng/μL. The concentration of GUT1-KM3 was 5.04 ng/μL.

### The target interaction fragments were captured by immunomagnetic beads

400 μL of 1 × Tween Washing Buffer (5 mM Tris-HCl (pH 7.5), 0.5 mM EDTA, 1 M NaCl, 0.05% Tween 20) was added into the new centrifuge tube, then it was added 50 μL of Dynabeads MyOne Streptavidin T1 reagent (Life technologies, 65602). We mixed them and placed on a magnetic stand for 3 min. The supernatant buffer was removed. We added 300 μL of 2 × Binding Buffer (10 mM Tris-HCl (pH 7.5), 1 mM EDTA, 2 M NaCl) to resuspend the magnetic beads, then added the library to the magnetic bead mixture. The solution was pipetted, mixed well, and rotated at RT for 15 min. We return the sample tube to the magnetic stand. After about 5 min, the solution became clear. The supernatant was discard. We added 600 μL of 1 × TWB, mixed well, and incubated at 55°C for 2 min on metal bath. Then we return them to the magnetic rack for 3 min, and discarded the supernatant. After 1 × TWB washing again and air dry the beads at RT for 3 min, 25 μL of ultrapure water was added to dissolve the beads with sample DNA. So that we obtained biotin-labeled joint DNA, which had been removed circular chaperone DNA. The subsequent steps were performed with immunomagnetic beads.

### End repair and A-tailing reactions was performed and the library was amplified and sequenced

Here, the input DNA solution is 25 μL of biotin-labeled DNA sample obtained in the previous step 1.8. In the end repair and A-tailing reaction process, the amount of related reagent is halved 7.5 μL of End Prep Mix4 (Vazyme ND607) was add in the input DNA solution. We vortexed them gently, transferred to a PCR machine and incubated immediately (20°C for 15 min, 65°C for 15 min, and 4°C ∞) to obtain the end repair and A-tailing products. Then the products were added the adapter. We prepared the adapter ligation mixture (32.5 μL of end repair and A-tailing reaction product, 1 μL of Adapter stock solution (Vazyme ND805), 1.5 μL of ultrapure water, 12.5 μL of Rapid Ligation buffer, and 2.5 μL of Rapid DNA ligase). They were mixed well and centrifuged gently. For quickly connect the adapter, they were incubated at 20°C for 15 min. Then we washed the sample beads. We added 150 μL of 1 × TE buffer, mixed well, and washed twice. Then we also added 150 μL of 1 × B/W buffer, mixed well, and washed twice. Finally, we added 40 μL ultrapure water to dissolve the beads. Next, we performed PCR-QC pre-amplification. PCR-QC mixture (10 μL of 2 × HiFi HotStart Ready Premix (KAPA Biosystems, KK8502), 1 μL of 10 × Library Amplification Primer Master Mix, 1 μL of the obtained product after adapter adding, and 8 μL of ultrapure water) was prepared and thoroughly mixed. PCR program was set up as follows: pre-denaturation at 98°C for 1 min, 12 or 18-cycle amplification (denaturation at 98°C for 15 s, annealing at 60°C for 30 s, and extension at 72°C for 30 s), and final extension at 72°C for 5 min, and storage at 4°C ∞ min. Thus, PCR products were obtained. After instantaneous centrifugation, we pipetted 19 μL PCR-QC product for 2% E-gel detection. The result is shown in Figure S1B, which indicates that quality control of DNA library is successful.

According to PCR-QC result, we settled the amplification cycle of formal PCR is ~6. After PCR, magnetic bead purification is performed. In detail, we added Ampure XP beads (the volume ratio is 1 to 1) into the formal PCR reaction solution, mixed well, and stood at RT for 5 min. Then it was placed on the magnetic rack for 5 min, after which the solution became clear, and the supernatant was discarded. We washed the beads twice with 500 μL of 80% ethanol, and dried for 3 min. 20 μL of ultrapure water was added to dissolve, and mixed well. They were stood at RT for 5 min and returned to the magnetic rack for 5 min. The supernatant was transferred to a new centrifuge tube.

For library size selection, the purified formal PCR library (~20 μL) was run on E-gel, and the fragments with size of 350~700 bp were recovered by Gel Recovery Kit (Zymoclean™, D4008), which was the final GutHi-C sequencing library. The results were shown in Figure S1C. The library (19 μL of the volume) recovery concentrations were 1.46 ng/μL (GUT1-KM1), 2.4 ng/μL (GUT1-KM2) and 4.8 ng/μL (GUT1-KM3), respectively. In this research, we pipetted 10 ng of each sample senting for sequencing. All samples were mixed according to the mass ratio of 1 to 1, and then subjected to pre-operation quality control. Real-time-qPCR and NGS 3K library quality detection was performed. Then the DNA library sample was submitted to Illumina NovaSeq 6000 or NovaSeq X Plus PE150 paired-end sequencing platform.

## Supplemental Result

### Data evaluation and result analysis for GutHi-C libraries, which were sequenced in small size data

For HiC-Pro result (here, the second-generation sequencing assembled chicken gut metagenome is as reference genome input), the mapping rate refers to the unique matching ratio across three GutHi-C samples, which was calculated as the ratio of mapped reads (the numerator) to the total number of reads (the denominator). This metric provides an assessment of the efficiency of reads mapping. Results depicted in Figure 1B highlight GutHi-C's superiority in average matching rates across the three samples and within respective treatment groups. Additionally, the valid data output rates (Valid/Total) were examined to assess the ratio of effective data (contains no PCR duplication) to total sequencing data. It is demonstrated that GutHi-C has higher effectiveness. Furthermore, as shown in Figure 1B, regarding the comparison of the valid interaction pair proportion (valid interaction pairs/unique paired alignments). GutHi-C demonstrates its superior data quality. Here, valid interaction pairs represent the counts of valid proximal interaction pairs, including those before the removal of PCR duplicates in the library preparation. Besides, GutHi-C exhibits lower multiple pairs alignment rate and unmapped pairs rate.

Comprehensively, as known, it is required for HiC-Pro analysis to input reference genome (Here, referring to the metagenome). Due to limitations in the metagenome assembly quality of second-generation Shotgun sequencing (also known as next generation sequencing), the resulting microbial genome after assembly is highly fragmented. To better utilize this GutHi-C technique to demonstrate genome-wide interactions information of microorganisms at the single-bacteria resolution level, we replaced second-generation sequencing assembled gut metagenome with third-generation sequencing assembled gut metagenome, when analyzing the microbial genome interaction frequency. In detail, we utilized previously obtained third-generation sequencing assemblled chicken gut metagenome by Zhang et al. as a reference genome [39]. As the gaps in the second-generation assembled metagenome were filled by the third-generation sequencing, it normally lead to a decrease in GutHi-C mapping rates. Nevertheless, the maping rates of GutHi-C remained superior to the control group [25]. The mapping rate of our method is ~17.27%, while the control group is ~13.67%. More comprehensively, we downloaded two Hi-C datasets and assembled genome information from bovine rumen samples (NCBI PRJEB21624). Subsequently, we analyzed this dataset using HiCPro V3.1. The valid interaction pair proportions and mapping rates for Cow1 and Cow2 are consistent with the Hi-C results for sheep [25]. According to the Hi-C library construction methods described in the two articles, the same library construction method was employed (also using ProxiMeta Hi-C). However, the results are significantly lower than the valid pair ratio observed in the GutHi-C (GUT1 series) [28].

### Big data size sample assessment analysis and preliminary application to reveal the 3D conformation of microbial metagenomes

In this section of the research, we have re-collected the gut microbiota of the experimental chickens and reconstructed the GutHi-C libraries, which were submitted to large-scale sequencing (approximately 100~150 gigabases of raw data). As illustrated in Figure S2 below, there are three experimental condition variables set here. The first condition variable includes Gut5-1, Gut5-2, and Gut5-A washed using LB medium; Gut5-D, Gut5-E, and Gut5-F rinsed with 10 mM MgSO_4_. For the second condition variable, Gut5-1 and Gut5-2 are handled by the first operator. Gut5-A, Gut5-D, Gut5-E, and Gut5-F are handled by the second operator. The third condition variable involves Gut5-F with microbial lysis just using lysozyme method, while in other samples (Gut5-1, Gut5-2, Gut5-A, Gut5-D, and Gut5-E), microbial lysis methods are included lysozyme and liquid nitrogen grinding.

The correlation among these groups falls within the range of 0.6~0.7, indicating that inter-group differences are within the same order of magnitude. The Hi-C matrix correlation ranges from sixty to seventy percent because of the inherent characteristics of microbial metagenomes and the limited amount of valid data we acquired for comparison. As variations between different operators are considered normal, and the differences observed between LB and MgSO₄ treatments are smaller than inter-operator variances, these distinctions are deemed negligible. It proves LB liquid medium serves rinsing function, equivalent in using 10mM MgSO₄. This also suggests excellent repeatability of the method.

## Supplemental Discussion

Traditional Hi-C protocols are primarily designed for mammalian cell lines. Although Hi-C technology has been adapted for use in mammalian tissues, plant tissues, and cellular levels, its application in microbial research is relatively limited, lacking mature and efficient microbial Hi-C protocols. Currently, the widely used ProxiMeta Hi-C [25,27−29,31,40] is so expensive and banned for sale in China, making it inaccessible through conventional compliant commerce channels.

Compared with the Hi-C of plant cells, microorganisms and plant cells are essentially different. There are at least 1000~1150 kinds of bacteria in the intestinal tract, and each host contains about 160 dominant species on average. Whether human or animal, the intestinal microbial population and quantity are extremely complex, and intestinal microorganisms not only have cell walls but also have the influence of sediment, inorganic salts, soil, plant residues, and other substances when they are lysed [16]. Although the Hi-C of plant cells discloses the method of ''optimizing the process of plant cells, quick-freezing and grinding the biological samples to be tested in liquid nitrogen [41]'', there are many ways to break cell walls. In this experiment, some tests have been carried out by us. The ''liquid nitrogen grinding and lysozyme'' treatment, being a collection processed by both ''grinding only by liquid nitrogen'' treatment and ''lysozyme only (without grinding)'' treatment, may contain a few of fragmented DNA, which potentially reduces its alignment rate (mapping rate and multi-mapping rate) and marginally increases the unaligned rate (unmapped pairs rate). However, for valid data, such an outcome is considered acceptable. Optimizing each step can reduce the proportion of dangling end, improve the proportion of valid data and decrease ''interbacterial-interaction'' noise. In the restriction endonuclease digestion step, it uses the ''four-site'' RE-enzyme DpnII, which improves the efficiency of digestion, increases the proportion of overhang fragments that can be used for proximity-ligation and has the potential to achieve higher resolution. Especially, a circular coprecipitation DNA control step is introduced, so that the DNA processing process can be visualized at low cell input, and the loss of the metagenomic DNA in the library building could be reduced. In the process of DNA ultrasonic fragmentation, the fragment size was subjected to magnetic bead sorting. As a result, we can get a higher purification consistency, we also can ensure better purification quality. Interestingly, we found that the cut-off recovery range of formal PCR E-gel results is more than 350 bp, which can reduce the probability of adapter contamination. These optimizations in the process of operation further round out the technology.

In the results section, we evaluated the feasibility and results of GutHi-C technology in detail. Mainly from the following aspects: First, data quality assessment. Second, effective data comparison. Third, the ability to reveal the three-dimensional genome interactions and conformations of microorganisms. Fourth, it is used to assist in the assembly of high-quality metagenomes of microorganisms. We evaluated and compared the small sequencing data level and the large sequencing data level, respectively, and proved the reliability and superiority of GutHi-C technology.

What needs to be explored here are TAD (Topologically Associating Domain) or Loop domians calling and related higher-order interactions disclosing, existing literature suggests that bacteria lack high dimensional conformation like TAD domains defined in eukaryotes and exhibit only simple interaction patterns [42]. However, through a series of GutHi-C technical experiments utilizing freshly collected chicken gut microbiota, including library construction and sequencing, we generated a substantial dataset of approximately 100~150 G of raw bases data for comparative analysis of this issue. Our results indicate there are regions within individual bacteria in the chicken gut microbiota that represent strong interactive patterns (Figure S3, and 2A-C), while in the published Hi-C heatmaps, there is no apparent presence of interaction domains. It could be stated that GutHi-C proves in a single microorganism, there are chromatin interaction domains (CIDs), similar to TADs in eukaryotes, exhibiting stronger interaction signals.

In the research, we developed the GutHi-C technology for microbial populations, which can maximize the dissociation of the microbial cell wall and allow the endonuclease to fully access the microbial genome. The amount of biotin and magnetic beads is reduced, which greatly reduces the cost. When performing high-throughput sequencing, the data HiC-Pro parameter evaluation, such as unique matching rate and effective data output rate, is better [25]. We also constructed some heatmaps of interaction frequencies for the GutHi-C data, which showed that intra-microorganism interaction frequency outperformed those of the ProxiMeta Hi-C under the same data level. GutHi-C also showed stronger CID or Loop domains. In terms of assembly application, about the most accurate three-generation sequencing platform-PacBio Revio HiFi sequencing, HiFi metagenome sequencing offers significant advantages, such as ~20 kb read length and > 99.9% accuracy for HiFi reads. However, with HiFi sequencing alone, there are still a certain number of microorganisms that are not fully assembled (loop-formed). It revealed that GutHi-C enables the classification of low-quality or medium-quality contigs, thereby enhancing the assembly quality of HiFi for high-quality metagenomes. Overall, GutHi-C improves sequencing data quality and reduces experimental costs, making it applicable to a wider range of applications.

## REFERENCES

1. Lieberman-Aiden, Erez, Nynke L. van Berkum, Louise Williams, Maxim Imakaev, Tobias Ragoczy, Agnes Telling, Ido Amit, et al. 2009. “Comprehensive mapping of long-range interactions reveals folding principles of the human genome.” *Science* 326: 289-293. <https://doi.org/10.1126/science.1181369>

2. Lesne, Annick, Julien Riposo, Paul Roger, Axel Cournac, Julien Mozziconacci. 2014. “3D genome reconstruction from chromosomal contacts.” *Nature Methods* 11: 1141-1143. <https://doi.org/10.1038/nmeth.3104>

3. Schmitt, Anthony D., Ming Hu, Bing Ren. 2016. “Genome-wide mapping and analysis of chromosome architecture.” *Nature Reviews Molecular Cell Biology* 17: 743-755. <https://doi.org/10.1038/nrm.2016.104>

4. Szałaj, Przemysław, Dariusz Plewczynski. 2018. “Three-dimensional organization and dynamics of the genome.” *Cell Biology and Toxicology* 34: 381-404. <https://doi.org/10.1007/s10565-018-9428-y>

5. Hnisz, Denes, Abraham Weintraub, Daniel Day, Anne-Laure Valton, Rasmus Bak, Charles Li, Johanna Goldmann, et al. 2016. “Activation of proto-oncogenes by disruption of chromosome neighborhoods.” *Science (New York, N.Y.)* 351: 1454-1458. <https://doi.org/10.1126/science.aad9024>

6. Korbel, Jan O., Charles Lee. 2013. “Genome assembly and haplotyping with Hi-C.” *Nature Biotechnology* 31: 1099-1101. <https://doi.org/10.1038/nbt.2764>

7. Burton, Joshua N., Andrew Adey, Rupali P. Patwardhan, Ruolan Qiu, Jacob O. Kitzman, Jay Shendure. 2013. “Chromosome-scale scaffolding of de novo genome assemblies based on chromatin interactions.” *Nature Biotechnology* 31: 1119-1125. <https://doi.org/10.1038/nbt.2727>

8. Liu, Chang. 2017. “In situ Hi-C library preparation for plants to study their three-dimensional chromatin interactions on a genome-wide scale.” Methods Mol Biol 1629: 155-166. <https://doi.org/10.1007/978-1-4939-7125-1_11>

9. Nagano, Takashi, Yaniv Lubling, Tim J. Stevens, Stefan Schoenfelder, Eitan Yaffe, Wendy Dean, Ernest D. Laue, Amos Tanay, Peter Fraser. 2013. “Single-cell Hi-C reveals cell-to-cell variability in chromosome structure.” *Nature* 502: 59-64. <https://doi.org/10.1038/nature12593>

10. Dryden, Nicola, Laura Broome, Frank Dudbridge, Nichola Johnson, Nick Orr, Stefan Schoenfelder, Takashi Nagano, et al. 2014. “Unbiased analysis of potential targets of breast cancer susceptibility loci by Capture Hi-C.” *Genome research* 24: 1854-1868. <https://doi.org/10.1101/gr.175034.114>

11. Ma, Wenxiu, Ferhat Ay, Choli Lee, Gunhan Gulsoy, Xinxian Deng, Savannah Cook, Jennifer Hesson, et al. 2018. “Using DNase Hi-C techniques to map global and local three-dimensional genome architecture at high resolution.” *Methods* 142: 59-73. [https://doi.org/https://doi.org/10.1016/j.ymeth.2018.01.014](https://doi.org/https:/doi.org/10.1016/j.ymeth.2018.01.014)

12. Li, Wen, Jiansen Lu, Ping Lu, Yun Gao, Yichen Bai, Kexuan Chen, Xinjie Su, et al. 2023. “scNanoHi-C: a single-cell long-read concatemer sequencing method to reveal high-order chromatin structures within individual cells.” *Nature Methods* 20: 1493-1505. <https://doi.org/10.1038/s41592-023-01978-w>

13. Maslova, A., V. Plotnikov, M. Nuriddinov, M. Gridina, V. Fishman, A. Krasikova. 2023. “Hi-C analysis of genomic contacts revealed karyotype abnormalities in chicken HD3 cell line.” *BMC Genomics* 24: 66-79. <https://doi.org/10.1186/s12864-023-09158-y>

14. Miao, Yuanxin, Yunxia Zhao, Siqi Wan, Quanshun Mei, Heng Wang, Chuanke Fu, Xinyun Li, Shuhong Zhao, Xuewen Xu, Tao Xiang. 2023. “Integrated analysis of genome-wide association studies and 3D epigenomic characteristics reveal the BMP2 gene regulating loin muscle depth in Yorkshire pigs.” PLoS Genetics 19: e1010820. <https://doi.org/10.1371/journal.pgen.1010820>

15. Sandoval-Velasco, Marcela, Juan Antonio Rodríguez, Cynthia Perez Estrada, Guojie Zhang, Erez Lieberman Aiden, Marc A. Marti-Renom, M. Thomas P. Gilbert, Oliver Smith. 2020. “Hi-C chromosome conformation capture sequencing of avian genomes using the BGISEQ-500 platform.” *GigaScience* 9: giaa087. <https://doi.org/10.1093/gigascience/giaa087>

16. Wang, Wenjing., Longjian. Niu, Chunhui. Hou. 2022. “Interrogating global chromatin interaction network by high-throughput chromosome conformation capture (Hi-C) in plants.” Methods in Molecular Biology (Clifton, N.J.) 2484: 55-67.

17. Yang, Tingting, Dingyue Wang, Guangmei Tian, Sun Linhua, Minqi Yang, Xiaochang Yin, Jun Xiao, et al. 2022. “Chromatin remodeling complexes regulate genome architecture in Arabidopsis.” The Plant Cell 34: 2638-2651. <https://doi.org/10.1093/plcell/koac117>

18. Dong, Qianli, Ning Li, Xiaochong Li, Zan Yuan, Dejian Xie, Xiaofei Wang, Jianing Li, et al. 2018. “Genome‐wide Hi‐C analysis reveals extensive hierarchical chromatin interactions in rice.” *The Plant Journal* 94: 1141-1156. <https://doi.org/10.1111/tpj.13925>

19. Han, Jin, Siyuan Wang, Wu Hongyu, Zhao Ting, Xueying Guan, Lei Fang. 2023. “An upgraded method of high-throughput chromosome conformation capture (Hi-C 3.0) in cotton (Gossypium spp.).” *Frontiers in Plant Science* 14: 1223591-1223607. <https://doi.org/10.3389/fpls.2023.1223591>

20. Davoodi, Saeideh, Edan Foley. 2020. “Host-microbe-pathogen interactions: a review of vibrio cholerae pathogenesis in drosophila.” *Frontiers in Immunology* 10: 3128-3138. <https://doi.org/10.3389/fimmu.2019.03128>

21. Yeoman, Carl, Nicholas Chia, Patricio Jeraldo, Maksim Sipos, Nigel Goldenfeld, Bryan White. 2012. “The microbiome of the chicken gastrointestinal tract.” *Animal health research reviews / Conference of Research Workers in Animal Diseases* 13: 89-99. <https://doi.org/10.1017/S1466252312000138>

22. Oakley, Brian B., Hyun S. Lillehoj, Michael H. Kogut, Woo K. Kim, John J. Maurer, Adriana Pedroso, Margie D. Lee, Stephen R. Collett, Timothy J. Johnson, Nelson A. Cox. 2014. “The chicken gastrointestinal microbiome.” *FEMS Microbiology Letters* 360: 100-112. <https://doi.org/10.1111/1574-6968.12608>

23. Glendinning, Laura, Robert D. Stewart, Mark J. Pallen, Kellie A. Watson, Mick Watson. 2020. “Assembly of hundreds of novel bacterial genomes from the chicken caecum.” *Genome Biology* 21: 34-49. <https://doi.org/10.1186/s13059-020-1947-1>

24. Zhou, Saisai, Runbo Luo, Ga Gong, Yifei Wang, Zhuoma Gesang, Kai Wang, Zhuofei Xu, Sizhu Suolang. 2020. “Characterization of metagenome-assembled genomes and carbohydrate-degrading genes in the gut microbiota of tibetan pig.” *Frontiers in Microbiology* 11: 595066-595076. <https://doi.org/10.3389/fmicb.2020.595066>

25. Bickhart, Derek M., Mikhail Kolmogorov, Elizabeth Tseng, Daniel M. Portik, Anton Korobeynikov, Ivan Tolstoganov, Gherman Uritskiy, et al. 2022. “Generating lineage-resolved, complete metagenome-assembled genomes from complex microbial communities.” *Nature Biotechnology* 40: 711-719. <https://doi.org/10.1038/s41587-021-01130-z>

26. Kim, Chan Yeong, Junyeong Ma, Insuk Lee. 2022. “HiFi metagenomic sequencing enables assembly of accurate and complete genomes from human gut microbiota.” *Nature Communications* 13: 6367-6377. <https://doi.org/10.1038/s41467-022-34149-0>

27. Gounot, Jean-Sebastien, Minghao Chia, Denis Bertrand, Woei-Yuh Saw, Aarthi Ravikrishnan, Adrian Low, Yichen Ding, et al. 2022. “Genome-centric analysis of short and long read metagenomes reveals uncharacterized microbiome diversity in Southeast Asians.” *Nature Communications* 13: 6044-6054. <https://doi.org/10.1038/s41467-022-33782-z>

28. Stewart, Robert D., Marc D. Auffret, Amanda Warr, Andrew H. Wiser, Maximilian O. Press, Kyle W. Langford, Ivan Liachko, et al. 2018. “Assembly of 913 microbial genomes from metagenomic sequencing of the cow rumen.” *Nature Communications* 9: 870-880. <https://doi.org/10.1038/s41467-018-03317-6>

29. Bickhart, Derek M., Mick Watson, Sergey Koren, Kevin Panke-Buisse, Laura M. Cersosimo, Maximilian O. Press, Curtis P. Van Tassell, et al. 2019. “Assignment of virus and antimicrobial resistance genes to microbial hosts in a complex microbial community by combined long-read assembly and proximity ligation.” *Genome Biology* 20: 153-170. <https://doi.org/10.1186/s13059-019-1760-x>

30. Stewart, Christopher J., Nadim J. Ajami, Jacqueline L. O’Brien, Diane S. Hutchinson, Daniel P. Smith, Matthew C. Wong, Matthew C. Ross, et al. 2018. “Temporal development of the gut microbiome in early childhood from the TEDDY study.” *Nature* 562: 583-588. <https://doi.org/10.1038/s41586-018-0617-x>

31. Stalder, Thibault, Maximilian O. Press, Shawn Sullivan, Ivan Liachko, Eva M. Top. 2019. “Linking the resistome and plasmidome to the microbiome.” *The ISME Journal* 13: 2437-2446. <https://doi.org/10.1038/s41396-019-0446-4>

32. Martin, Marcel. 2011. “Cutadapt removes adapter sequences from high-throughput sequencing reads.” EMBnet.journal 17: 10-12. <https://doi.org/10.14806/ej.17.1.200>

33. Servant, Nicolas, Nelle Varoquaux, Bryan R. Lajoie, Eric Viara, Chong-Jian Chen, Jean-Philippe Vert, Edith Heard, Job Dekker, Emmanuel Barillot. 2015. “HiC-Pro: an optimized and flexible pipeline for Hi-C data processing.” *Genome Biology* 16: 259-269. <https://doi.org/10.1186/s13059-015-0831-x>

34. Ramírez, Fidel, Vivek Bhardwaj, Laura Arrigoni, Kin Chung Lam, Björn A. Grüning, José Villaveces, Bianca Habermann, Asifa Akhtar, Thomas Manke. 2018. “High-resolution TADs reveal DNA sequences underlying genome organization in flies.” *Nature Communications* 9: 189-203. <https://doi.org/10.1038/s41467-017-02525-w>

35. DeMaere, Matthew Z., Aaron E. Darling. 2019. “bin3C: exploiting Hi-C sequencing data to accurately resolve metagenome-assembled genomes.” *Genome Biology* 20: 46-61. <https://doi.org/10.1186/s13059-019-1643-1>

36. Chklovski, Alex, Donovan H. Parks, Ben J. Woodcroft, Gene W. Tyson. 2023. “CheckM2: a rapid, scalable and accurate tool for assessing microbial genome quality using machine learning.” *Nature Methods* 20: 1203-1212. <https://doi.org/10.1038/s41592-023-01940-w>

37. Heinz, Sven, Christopher Benner, Nathanael Spann, Eric Bertolino, Yin C. Lin, Peter Laslo, Jason X. Cheng, Cornelis Murre, Harinder Singh, Christopher K. Glass. 2010. “Simple combinations of lineage-determining transcription factors prime cis-regulatory elements required for macrophage and B cell identities.” *Molecular Cell* 38: 576-589. [https://doi.org/https://doi.org/10.1016/j.molcel.2010.05.004](https://doi.org/https:/doi.org/10.1016/j.molcel.2010.05.004)

38. Rao, Suhas S P., Miriam H Huntley, Neva C Durand, Elena K Stamenova, Ivan D Bochkov, James T Robinson, Adrian L Sanborn, et al. 2014. “A 3D map of the human genome at kilobase resolution reveals principles of chromatin looping.” *Cell* 159: 1665-1680. [https://doi.org/https://doi.org/10.1016/j.cell.2014.11.021](https://doi.org/https:/doi.org/10.1016/j.cell.2014.11.021)

39. Zhang, Yan, Fan Jiang, Boyuan Yang, Sen Wang, Hengchao Wang, Anqi Wang, Dong Xu, Wei Fan. 2022. “Improved microbial genomes and gene catalog of the chicken gut from metagenomic sequencing of high-fidelity long reads.” *GigaScience* 11: giac116. <https://doi.org/10.1093/gigascience/giac116>

40. Press, Maximilian, Andrew Wiser, Zev Kronenberg, Kyle Langford, Migun Shakya, Chien-Chi Lo, Kathryn Mueller, Shawn Sullivan, Patrick Chain, Ivan Liachko. 2017. “Hi-C deconvolution of a human gut microbiome yields high-quality draft genomes and reveals plasmid-genome interactions.” *bioRxiv* <https://doi.org/10.1101/198713>

41. Grob, Stefan. 2022. Tough Tissue Hi-C. *Spatial Genome Organization: Methods and Protocols* Springer US, 35-50. <https://doi.org/10.1007/978-1-0716-2497-5_3>

42. Feng, Xu, Qihong Huang, Qunxin She. 2019. “Crenarchaeal 3D genome: a prototypical chromosome architecture for eukaryotes.” *Cell* 179: 56-58. [https://doi.org/https://doi.org/10.1016/j.cell.2019.08.045](https://doi.org/https:/doi.org/10.1016/j.cell.2019.08.045)

## Supplemental Figure


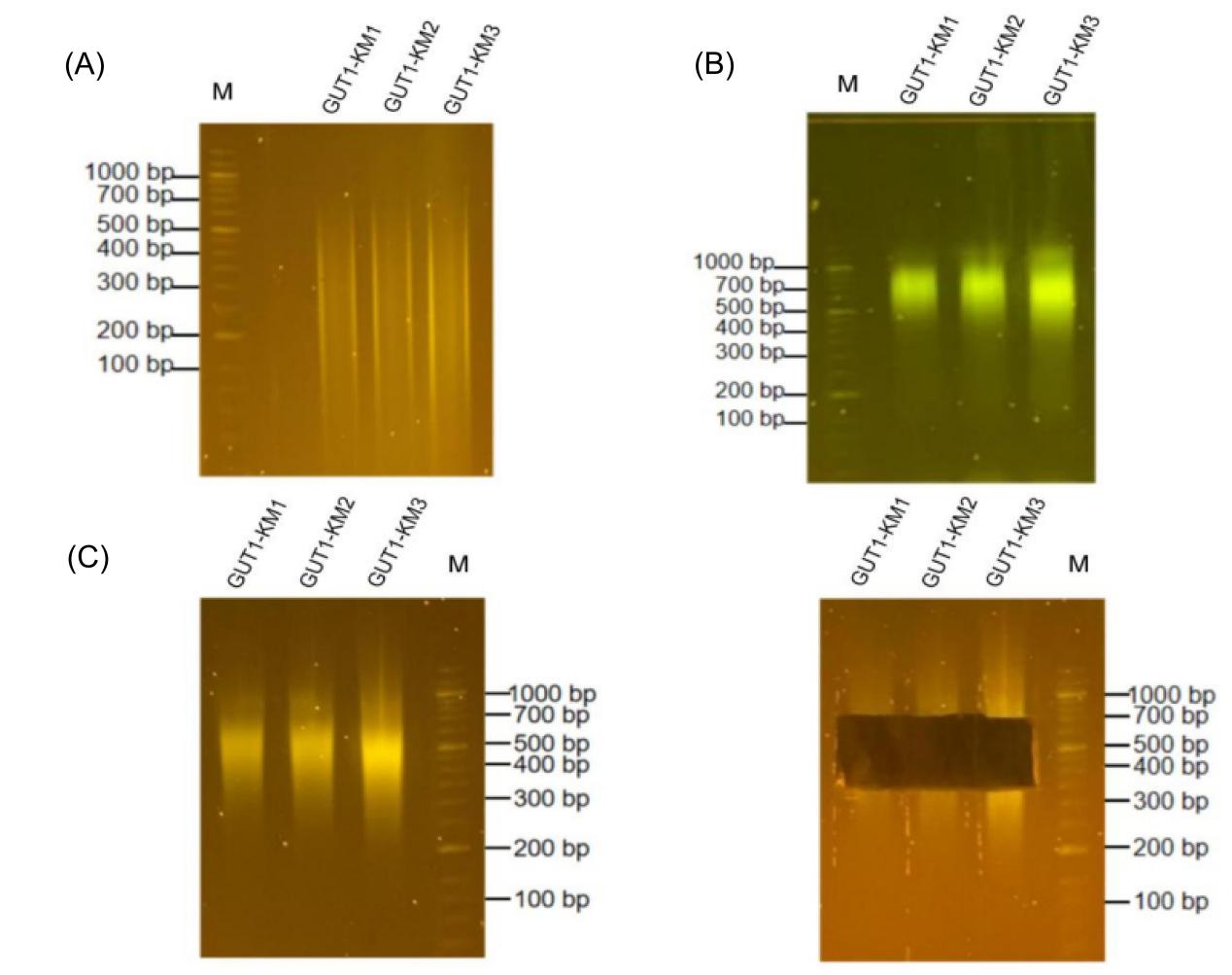


**Figure S1 Gel plots for the quality control during library construction. (**A) Gel images of the quality control of broken interacting DNA after sonication. Line 1 represents the Marker, also known as DNA ladder, ranging from 100 to 1000 bp. Line 3 represents GUT1-KM1, line 4 represents GUT1-KM2, and line 5 represents GUT1-KM3. (B) Gel images of the PCR-QC pre-experiments with 12 cycles. Line 1 represents the Marker, line 2 represents GUT1-KM1, line 3 represents GUT1-KM2, and line 4 represents GUT1-KM3. (C) Gel images of the formal PCR experiments. On the left, line 1 represents GUT1-KM1, line 2 represents GUT1-KM2, and line 3 represents GUT1-KM3. Line 4 represents the Marker. On the right, it is a picture after cutting the E-gel.


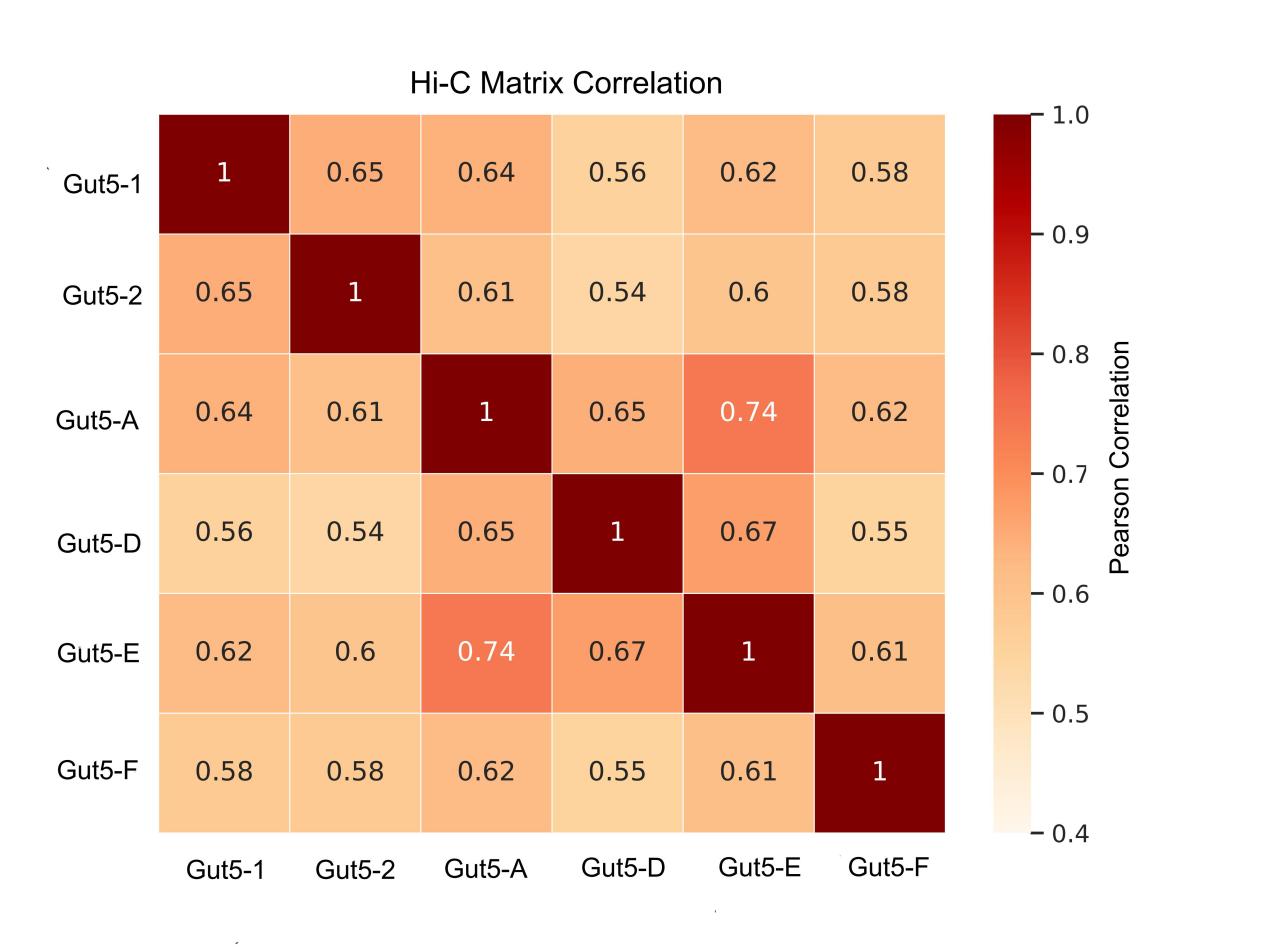


**Figure S2 Correlation of GutHi-C matrices under different experimental conditions.** Gut5-1: Experimental operations are performed by the first operator, rinsed the sample with LB medium, and subjected to microbial lysis methods including lysozyme and liquid nitrogen grinding; Gut5-2: Experimental operations are performed by the first operator, rinsed the sample with LB medium, and subjected to microbial lysis methods including lysozyme and liquid nitrogen grinding; Gut5-A: Experimental operations are performed by the second operator, rinsed the sample with LB medium, and subjected to microbial lysis methods including lysozyme and liquid nitrogen grinding; Gut5-D: Experimental operations are performed by the second operator, rinsed the sample with MgSO_4_, and subjected to microbial lysis methods including lysozyme and liquid nitrogen grinding; Gut5-E: Experimental operations are performed by the second operator, rinsed the sample with MgSO_4_, and subjected to microbial lysis methods including lysozyme and liquid nitrogen grinding; Gut5-F: Experimental operations are performed by the second operator, rinsed the sample with MgSO_4_, and subjected to microbial lysis with lysozyme only.


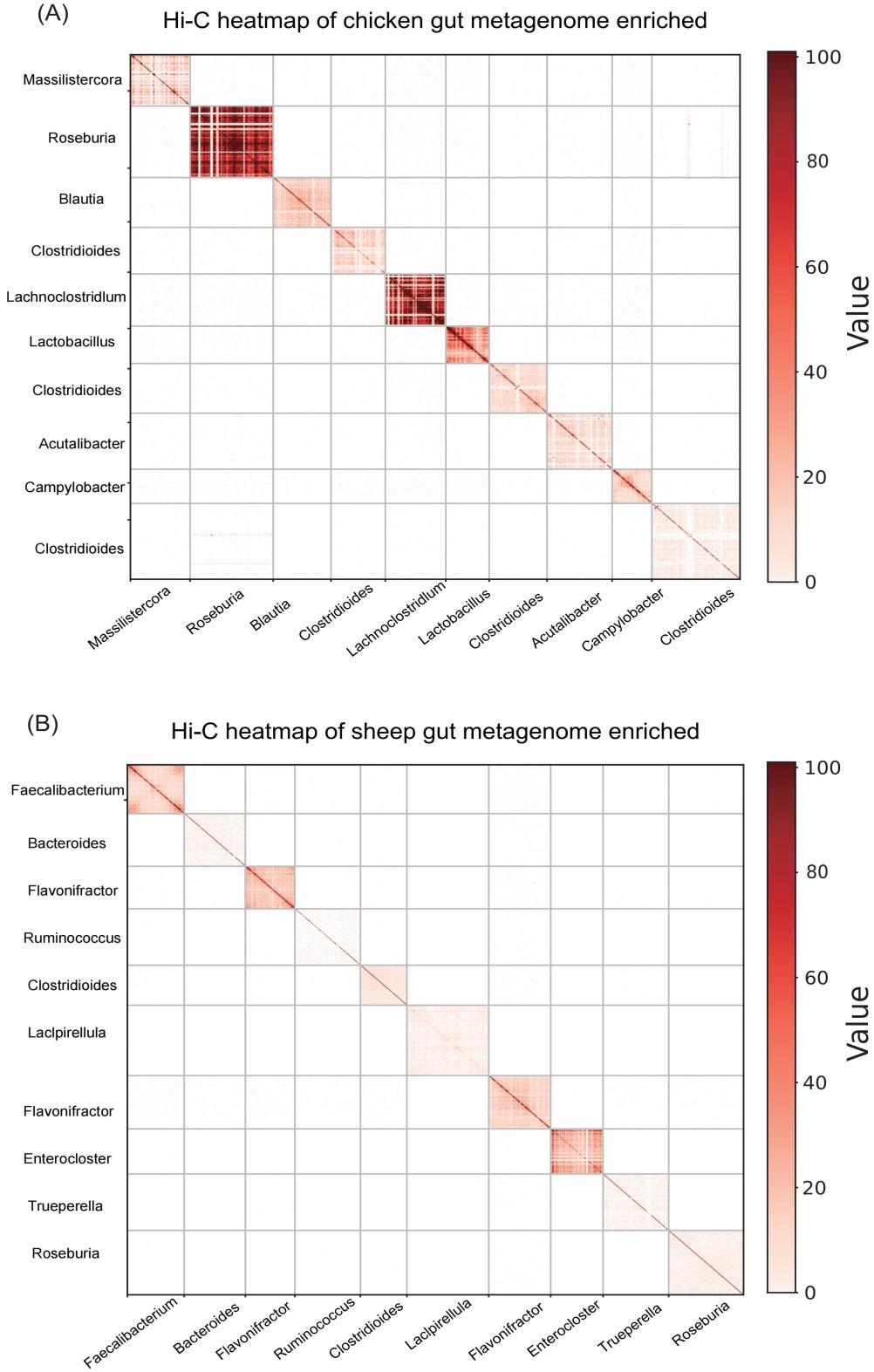


**Figure S3 Different resolution hierarchical structures of the single-bacterial metagenome revealed by GutHi-C, compared to ProxiMeta Hi-C.** (A) Hi-C heatmap of the top ten ranked single-bacterial metagenome captured by GutHi-C. (B) Hi-C heatmap of the top ten ranked single-bacterial metagenome captured by ProxiMeta Hi-C.
